# Lifestyle, APOE‐e4, and cognitive performance: a systematic review

**DOI:** 10.1002/alz70855_107097

**Published:** 2025-12-24

**Authors:** Dirce Samilly Souza da Silva, Mel Ferreira Silva Agnelo, Natalia Pozo Castro, Regina Silva Paradela, Naomi Vidal‐Ferreira

**Affiliations:** ^1^ Adventist College of Amazonia, Benevides, PA, Brazil; ^2^ Hospital San Borja Arriarán, Santiago, Region Metropolitana, Chile; ^3^ Global Brain Health Institute, Memory and Aging Center, University of California San Francisco, San Francisco, CA, USA; ^4^ University of Chile, Santiago, Region Matropolitana, Chile; ^5^ University of Chile, Santiago, Santiago, Chile; ^6^ University of São Paulo Medical School, São Paulo, São Paulo, Brazil; ^7^ Amazonia Adventist College, Benevides, PA, Brazil; ^8^ University of Sao Paulo Medical School, Sao Paulo, Brazil; ^9^ Adventist College of Amazonia, Benevides, Pará, Brazil

## Abstract

**Background:**

Dementia is a public health problem related to population aging. Alzheimer's disease, the main form of dementia, is associated with genetic factors, mainly with the e4 allele of the Apolipoprotein E (APOE‐e4), and environmental factors, such as lifestyle. However, the interaction of genetic and lifestyle factors in cognitive decline that leads to dementia is yet to be understood. Therefore, the aim of this study was to analyze the role of APOE‐e4 in the association of lifestyle interventions with cognitive performance using a systematic review.

**Method:**

Comprehensive searches were conducted in Pubmed (MEDLINE), in EMBASE, and in Web of Science. The eligibility criteria were: randomized clinical trial design, full‐text, lifestyle intervention, assessment of cognitive performance or dementia, and analysis of APOE‐e4. The last search was performed in May 2024. To conduct study analyses and inclusion as well as data extraction, the Covidence software was used.

**Result:**

Fifteen RCTs were included. Lifestyle interventions were mainly exercise, diet, or a combination of both. Six out of seven studies using exercise interventions observed cognitive improvements while three out of four studies using dietary interventions observed cognitive improvements in the whole sample. Even though all studies assessed APOE‐e4 status, only three studies compared the intervention effects between APOE‐e4 carriers and non‐carriers and in only one of them significant differences were observed.

**Conclusion:**

Our results show that lifestyle interventions such as exercise and diet might improve cognitive functions. However, the differences in patterns of lifestyle effects on APOE‐e4 carriers vs non‐carriers is still inconclusive. Future studies aiming at assessing possible differences between these conditions are needed.

